# Signatures of the exciton gas phase and its condensation in monolayer 1T-ZrTe_2_

**DOI:** 10.1038/s41467-023-36857-7

**Published:** 2023-02-27

**Authors:** Yekai Song, Chunjing Jia, Hongyu Xiong, Binbin Wang, Zhicheng Jiang, Kui Huang, Jinwoong Hwang, Zhuojun Li, Choongyu Hwang, Zhongkai Liu, Dawei Shen, Jonathan A. Sobota, Patrick Kirchmann, Jiamin Xue, Thomas P. Devereaux, Sung-Kwan Mo, Zhi-Xun Shen, Shujie Tang

**Affiliations:** 1 State Key Laboratory of Functional Materials for Informatics, Shanghai Institute of Microsystem and Information Technology, Chinese Academy of Sciences, Shanghai, PR China; 2 2020 X-Lab, Shanghai Institute of Microsystem and Information Technology, Chinese Academy of Sciences, Shanghai, PR China; 3 Stanford Institute for Materials and Energy Sciences, SLAC National Accelerator Laboratory, Menlo Park, CA USA; 4 Department of Physics, University of Florida, Gainesville, FL USA; 5 Geballe Laboratory for Advanced Materials, Departments of Physics and Applied Physics, Stanford University, Stanford, CA USA; 6 Key Laboratory for Power Machinery and Engineering of MOE, School of Mechanical Engineering, Shanghai Jiao Tong University, Shanghai, PR China; 7 School of Physical Science and Technology, ShanghaiTech University, Shanghai, PR China; 8 Advanced Light Source, Lawrence Berkeley National Laboratory, Berkeley, CA USA; 9 Department of Physics, Kangwon National Univerisity, Chuncheon, Korea; 10 Department of Physics, Pusan National University, Busan, Korea; 11 National Synchrotron Radiation Laboratory, University of Science and Technology of China, Hefei, Anhui PR China

**Keywords:** Electronic properties and materials, Two-dimensional materials, Surfaces, interfaces and thin films

## Abstract

The excitonic insulator (EI) is a Bose-Einstein condensation (BEC) of excitons bound by electron-hole interaction in a solid, which could support high-temperature BEC transition. The material realization of EI has been challenged by the difficulty of distinguishing it from a conventional charge density wave (CDW) state. In the BEC limit, the preformed exciton gas phase is a hallmark to distinguish EI from conventional CDW, yet direct experimental evidence has been lacking. Here we report a distinct correlated phase beyond the 2×2 CDW ground state emerging in monolayer 1T-ZrTe_2_ and its investigation by angle-resolved photoemission spectroscopy (ARPES) and scanning tunneling microscopy (STM). The results show novel band- and energy-dependent folding behavior in a two-step process, which is the signatures of an exciton gas phase prior to its condensation into the final CDW state. Our findings provide a versatile two-dimensional platform that allows tuning of the excitonic effect.

## Abstract (web summary)

Signatures of an excitonic insulator have been reported in several two-dimensional materials. Here the authors report electronic properties of monolayer ZrTe_2_ from ARPES and STM measurements that are consistent with the preformed exciton gas phase, a precursor for the excitonic insulator.

## Introduction

The macroscopic quantum phenomena such as superconductivity and BEC are crucial and attractive in basic research and potential technological applications. The EI was originally proposed as an analog of Bardeen-Cooper-Schrieffer (BCS) condensate in a weak-coupling limit^1–3^. It may support novel transport properties^4,5^ corresponding to the superfluidity in superconductors. With varying coupling strength, a wealth of exotic phenomena could stem from EIs^6,7^, such as correlated Chern insulator and fractionalized Hall effect^8^. However, the unequivocal experimental evidence for the realization of an EI state is still lacking. Unlike singlet-paired superconductors, the electron-hole pair in exciton does not carry a net charge, thus providing no easy observable physical quantity. Further complicating the issue, the EI state with finite-momentum exciton is characterized by a CDW state with periodic lattice distortion (PLD). Hardly any difference is present in the ground state electronic structure due to the same symmetry-breaking behavior in the electronically driven EI and a conventional CDW state from electron-phonon coupling (EPC).

Experimental efforts to identify the excitonic effect have been devoted to looking beyond the static electronic structure, such as the softening of the plasmon mode in BCS limit EI candidate^9^ and ultra-fast melting of the CDW order upon laser pumping^10^, or constructing van der Waals heterostructures and separating electron and hole layers to avoid the fast recombination^11,12^. Another distinct characteristic of excitonic condensate, in contrast to the conventional CDW state, is the existence of the exciton gas state in the BEC limit. However, due to the very limited candidate materials, the experimental observation of the exciton gas phase is still left to be explored^13^.

The EI candidate materials have been focused in the poorly screened quasi-low-dimensional materials, such as 2D material 1T-TiSe_2_^9,10,14^, monolayer 1 T′-WTe_2_^15,16^, quasi-1D material Ta_2_NiSe_5_^17,18^ and SmSe_0.45_Te_0.55_^19,20^ in which evidences of excitonic insulating state are reported. Notably, reduction of the dimensionality enhances the excitonic effect by suppressing the screening, as evidenced in the unprecedentedly strong excitonic effect of the monolayer transition metal dichalcogenides (TMDC)^15,16^ and other low dimensional materials^21–23^. The semi-metallic 1T-ZrTe_2_^24–27^, a sister compound of 1T-TiSe_2_, hosts a CDW state when thinning down to its 2D limit^28,29^. Compared to 1T-TiSe_2,_ the absence of CDW in bulk indicates the suppressed influence of EPC and a potential advantage in investigating the excitonic instability stemming from the characteristic band structure that nurtures the EI^2^.

In this work, using molecular beam epitaxy (MBE), we have successfully grown high-quality monolayer 1T-ZrTe_2_. In situ ARPES and STM measurements confirmed the emergence of a 2 × 2 CDW ground state in low temperature (LT)^28,29^, and revealed a strong spectral weight (SW) transfer and band folding. Using carrier doping to suppress the interaction, we uncovered the pristine electronic structure of the material (interaction-suppressed state). This insight is important to disclose that the high-temperature state (well above CDW transition temperature (T_c_)) is distinct from both the CDW and interaction-suppressed state. We call this novel high-temperature state the intermediate state. This two-step process of the CDW formation further reveals a novel band- and energy-dependent relaxation of the band folding. By comparing to the theoretical calculations, we conclude such observations decisively deviate from the conventional CDW systems, but naturally occur in the preformed exciton gas scenario^30^.

## Results

### Characterization of epitaxially grown monolayer 1T-ZrTe_2_

**Fig. 1:**
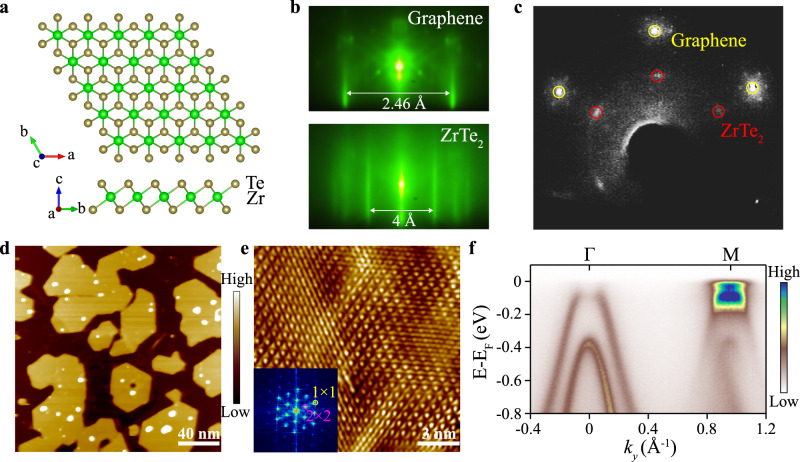
Structure and characterization of epitaxially grown monolayer 1T-ZrTe_2_. **a** Schematic crystal structure of 1T-ZrTe_2_. 1T-ZrTe_2_ in the top (top panel) and side (bottom panel) views. **b** RHEED patterns of graphene substrate (top panel) and sub-monolayer 1T-ZrTe_2_ (bottom panel). **c** LEED pattern after the film growing on the graphene. **d** STM image of monolayer 1T-ZrTe_2_ at 300 K [*V*_*bias*_ = −2.8 V, *I* = 200 pA, 200 nm × 200 nm]. Dark regions correspond to the BLG substrate, and the 1T-ZrTe_2_ layer is orange, which is in the form of islands with straight boundaries, dressed with a small number of particles that might be amorphous ZrTe compounds or tiny 1T-ZrTe_2_ islands. Some 1T-ZrTe_2_ islands with a corner angle of 120° are identified, implying a hexagonal symmetry of 1T-ZrTe_2_ single crystal. **e** Atomically resolved STM image of a CDW-related 2 × 2 superstructure at 4.5 K [*V*_*bias*_ = −50 mV, *I* = 200 pA, 15 nm × 15 nm]. The insert is the corresponding FFT. **f** ARPES measured band structure along the Γ-M direction at *T* = 15 K.

Figure 1a represents the crystal structure of the monolayer 1T-ZrTe_2_, where two Te layers sandwich a Zr layer with octahedral coordination. Sub-monolayer 1T-ZrTe_2_ were grown on bilayer graphene (BLG) terminated SiC substrate, which is known to induce the least amount of interaction between the MBE grown film and substrate^31,32^. Figure 1b, c show the reflection high-energy electron diffraction (RHEED) and low energy electron diffraction (LEED) images of the film, respectively, showing the hexagonal symmetry of the 2D crystal with the in-plane orientation aligned with the BLG substrate. Using the lattice constant of BLG (a = 2.46 Å) as a reference, the lattice constant of the film is estimated to be 4.0 ± 0.2 Å, consistent with the value of bulk 1T-ZrTe_2_. The large-scale STM image at room temperature in Fig. 1d shows the typical morphology of monolayer 1T-ZrTe_2_ domains, which are dispersed over the BLG substrate without forming a continuous monolayer film. The typical size of the monolayer domains is ~50 nm and the average coverage is around 70%. The ARPES data of monolayer 1T-ZrTe_2_ were obtained from the sub-monolayer films to avoid the signals from multilayer domains.

At T = 4.5 K, a 2 × 2 superlattice is observed by an atomically resolved STM image (Fig. 1e), and its 2D Fast Fourier transform (FFT) image (the insert in Fig. 1e), indicates the occurrence of CDW order at LT. In addition to the CDW order, long-range modulation, i.e., light-dark regions, could be the inhomogeneous doping from substrate. ARPES spectra (Fig. 1f) taken from the monolayer 1T-ZrTe_2_ along the Γ-M direction further confirm the existence of the CDW state. For the 2 × 2 CDW state, the first and second valence bands below Fermi energy (E_F_) at the Γ point are folded into the M point. The SW transfer is distinctive in the system: the SW of the low energy valence band at the Γ point is nearly depleted around its maximum, with most of the SW transferred to the folded band at the M point, while the SW of the conduction band at the M point is kept nearly intact close to E_F_. Notably, such observations were also made in bulk 1T-TiSe_2_, attributed to the formation of the EI^14,33^, which provides a corresponding reference to consider monolayer 1T-ZrTe_2_ as an EI candidate material.

### Temperature evolution of CDW state

**Fig. 2:**
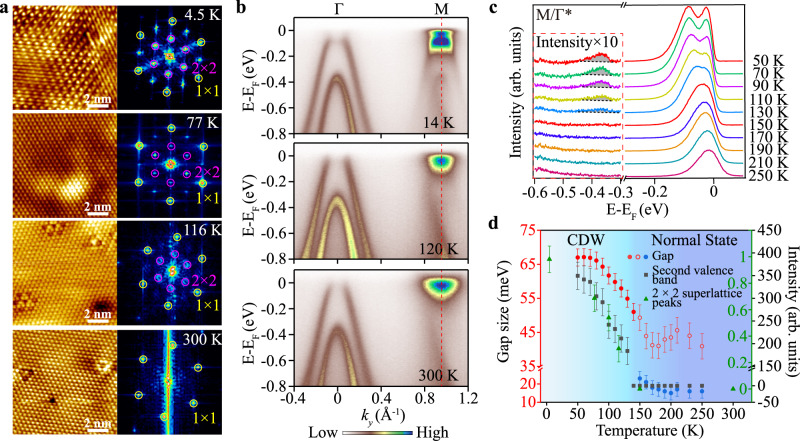
Temperature dependence of CDW state in monolayer 1T-ZrTe_2_. **a** Atomically resolved STM images of 10 nm × 10 nm and corresponding FFT images at 4.5 K [*V*_*bias*_ = −50 mV, *I* = 200 pA], 77 K [*V*_*bias*_ = 50 mV, *I* = 250 pA], 116 K [*V*_*bias*_ = −150 mV, *I* = 280 pA] and 300 K [*V*_*bias*_ = −100 mV, *I* = 300 pA]. **b** The band structures measured along the experimental Γ-M direction at 14, 120, and 300 K. **c** EDCs measured exactly at M/Γ* (correspond to red dashed lines in (**b**), as a function of temperature. Three peaks are clearly recognized in the spectra measured at *T* = 50 K, corresponding to one conduction band and two folded valence bands, respectively. **d** The evolutions of the size of the bandgap, the intensity of the folded second valence band, and 2 × 2 superlattice peaks in STM with temperature. At the high-temperature region, it is hard to tell whether the folded first valence band merges into the conduction band or disappears. Different fitting strategies (circles with different colors, Supplementary Fig. 2) are employed, and the results show an identical trend. The error bars indicate the standard error of the fit coherence times.

The temperature evolution of the CDW state in monolayer 1T-ZrTe_2_ was studied using STM and ARPES. Figure 2a shows the melting of the CDW order for selected temperatures from T = 4.5 K to T = 300 K. With increasing temperature, the superlattice contrast in the real space gets blurry (77 K) and then hardly visible (116 K). The 2 × 2 superlattice peaks in the FFT get weaker and diffused accordingly. Above some critical temperature, the superlattice peaks disappear, and only the Bragg peaks can be observed (a typical result at 300 K is shown in Fig. 2a). The melting of the CDW order is also characterized in ARPES by the bandgap (defined by the energy difference between the conduction and folded valence bands) shrinking and the disappearance of the folded second valence bands at the M point (Fig. 2b, c). As temperature increases, the folded first valence band gradually shifts to lower binding energy and merges with the conduction band. The folded second valence peak also slightly moves towards E_F_, and its SW (shaded area) in Fig. 2c gradually fades away up to T = 150 K. Note the presence of another feature from −0.2 eV to −0.8 eV that persists in all temperatures. This is likely related to the existence of the impurity phase or defect states^34^, which is not relevant to the current discussion.

The temperature evolution of bandgap size, the intensity of the folded second valence band, and the intensity of the normalized 2 × 2 superlattice peak are plotted in Fig. 2d. These three observables are indicators of the CDW order, showing the identical temperature evolution. The T_c_ in the monolayer 1T-ZrTe_2_ is thus determined to be about 130 ± 20 K. A detailed look into the valence band at 300 K (Supplementary Fig. 3) reveals that the top of the first valence band is well below E_F_. Surprisingly, a strong SW transfer of the first valence band, i.e., the depletion of SW at the Γ point, persists well above T_c_ as in the 300 K data of Fig. 2b, which does not follow the same trend as the other three indicators. Such asynchronous behavior of the first valence band implies the deviation from the conventional CDW picture where band modification and CDW formation goes hand in hand.

### Interaction-suppressed state

**Fig. 3:**
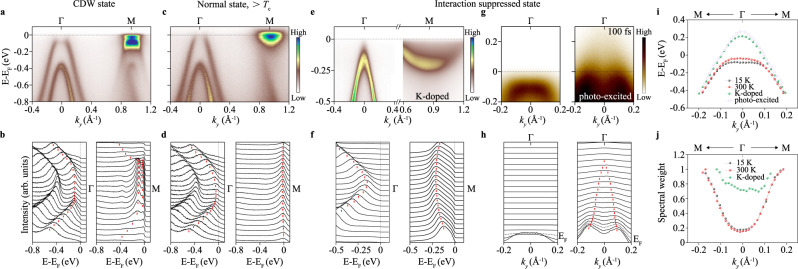
The two-step process of the CDW formation in monolayer 1T-ZrTe_2_. **a**, **c** The band structures measured by ARPES along the Γ-M direction at 15 K (**a**) and 300 K (**c**) using 50.6 eV synchrotron radiation light. **e** ARPES data along the Γ-M direction taken from surface K-doped sample with a carrier density of 0.14 e^−^/Zr atom. The corresponding EDCs are shown in (**b**, **d**, **f**) respectively. **g** ARPES snapshots taken before optical pumping and at characteristic pump-probe delays at the Γ point. **h** MDCs for the data shown in (**g**). **i**, **j** Band position (bands are shifted by aligning the second valence band to 15 K data for comparison) (**i**) and spectral weight (normalized by dividing by the maximum spectral weight) (**j**) of the first valence band at the Γ point along Γ-M direction. There is no apparent difference in the band dispersions and SW of the first valence band between the CDW (15 K) state and the normal state (300 K). In contrast, the interaction-suppressed state (photo-excited and K-doped) shows considerable band renormalization and SW recovery, respectively.

To investigate the nature of the asynchronous band folding, we use carrier doping to restore the screening in monolayer^24^ and suppress the many-body interaction^35,36^. Upon surface K (potassium)-doping^37^, the CDW order decreases drastically and eventually dissolves completely (Fig. 3e). Compared to the CDW state, a prominent valence band modification, from a flat band top to a sharp band dispersion, significantly shrinks the bandgap from ~70 meV to −40 meV, with a concomitant recovery of SW at the Γ point. To rule out the potential band distortion that K-doping may introduce^38^, we also employed photo-excitation with laser pulses to inject the carrier into the system to mimic the process of screening building up. The band dispersions before (*t* = −5 ps) and after (*t* = 100 fs) pumping at 12 K are plotted in Fig. 3g, exhibiting a transformation from a flattened valence band reshaped to a nearly linear dispersion upon pumping at the Γ point, which is identical to the K-doped case. The carrier injection suppresses the many-body effect and leads to an interaction-suppressed state, which is corroborated by reproducing the main electronic features of the noninteracting band structure from DFT calculations (Supplementary Fig. 4).

The direct experimental confirmation of the noninteracting state is especially important, which enables the disclosure of the existence of a correlated state prior to the CDW state. Comparing to the well screened state, the first valence band dispersion of the state at 300 K flattened prominently, which leads to a global bandgap opening and an accompanied strong SW transfer (Fig. 3i, j). In contrast, the STM measurement at 300 K does not show corresponding periodic lattice distortion (Fig. 2a). Such an discrepancy is the indications that there are strong phase fluctuations originate from the strong coupled nature of the material^39^, which is further proved by the relatively large Δ/T_c_ value. In which case, ARPES measures a non-zero gap (non-zero amplitude of order parameter), while the STM measurements of the CDW are averaged out by dynamic fluctuations.

## Discussion

**Fig. 4:**
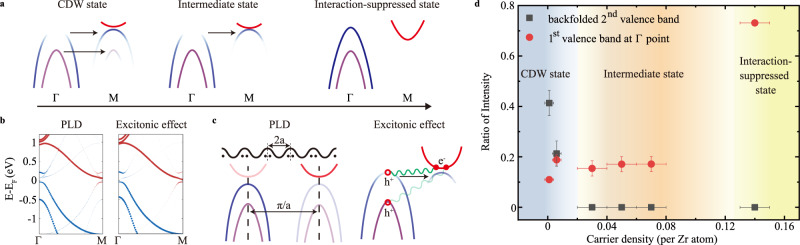
The origin of CDW phase in monolayer 1T-ZrTe_2_. **a** The schematic band diagram of the formation of the CDW state. **b** The calculated band structure along the Γ-M direction for CDW driven by PLD and excitonic effect, respectively. **c** The schematic diagram of PLD and excitonic effect, respectively. **d** The evolution of electronic structure features of CDW state with doping amount. The carrier density error bars represent the standard deviation of uncertainty in the calculation of the carrier doping amount. The ratio of intensity error bars represent the standard error of the fit coherence times.

As summarized in a schematic band diagram (Fig. 4a), the CDW formation is a two-step process, which can be attributed to the CDW order fluctuation. However, the second valence band folding occurs only in the CDW state, indicating a different driving force from the first valence band folding. In the following, we investigate the different roles that played by the candidate interactions, PLD and excitonic effect, in driving the band folding by the model calculations based on the microscopic Hamiltonians obtained from first-principles calculations, as shown in Fig. 4b (see Methods). For the PLD case, the folded SW from the first two valence bands with similar orbital components are both prominent^40^ (Fig. 4b left and 4c left), which agrees well with the experimentally observed LT CDW state. While for the exciton gas state, the excitonic interaction is much more energy- and band-dependent, making the band folding concentrated on the top of the first valence band and the bottom of the conduction band (Fig. 4b right and 4c right), which is consistent with the experimentally observed intermediate state.

Thus, the excitonic effect plays an indispensable role at the high-temperature intermediate state. Considering the absence of the long-range order in the intermediate state, it is a signature of the existence of the exciton gas phase. Further, the experimentally observed CDW fluctuation and the asynchronous band folding behavior occur naturally in BEC type of the exciton condensation. Above the BEC transition temperature T_c_, the excitonic effect hybridizes and renormalizes the valence and conduction bands^30^, and the preformed exciton is characterized by a folded valence band as seen in photoemisson^13^. Below T_c_, the exciton condensation drives a long-range CDW order formation, with PLD as secondary effect^33^. On top of the purely electronically driven first valence folding, a second valence band folding emerges upon the CDW formation. It should be noted that the observed PLD in the LT CDW state may not be solely driven by excitonic effect, the EPC could also play a key role.

In addition to the distinct temperature dependence of the two valence band foldings, an identical two-step process with the asynchronous band folding is also found by changing the doping amount, as indicated in the plot of the SWs of the first valence band top at the Γ point and the folded second valence band top at the M point as a function of carrier density amount in Fig. 4d. In which, the SW transfer of the second valence band, the indicator of the long-range CDW formation, is found to be very sensitive to the doping level. A critical doping amount of 2% electron per unit cell could suppress the SW transfer, which is also consistent with the expectation of the excitonic instability induced CDW^37^.

In summary, our experimental observation of the exciton gas phase in the monolayer 1T-ZrTe_2_ establishes it as a promising EI candidate material with a highly tunable electronic structure. Moreover, its layered nature is advantageous in constructing van der Waals heterostructures, which is promising in exploiting excitonic physics, such as realizing spin supercurrent by embracing magnetism^41^.

## Methods

### Film growth and STM measurements

The monolayer 1T-ZrTe_2_ films were grown by MBE on bilayer graphene (BLG) epitaxially grown on 4H-SiC. The base pressure of the system was ~5 × 10^−10^ mbar. Zr (99.95%) and Te (99.999%) were evaporated from an electron beam evaporator and thermal cracker cell, respectively. The substrate temperature was held at 330 °C during growth. The growth process was monitored by RHEED. After growth, the samples were transferred under vacuum to STM (VT, Omicron) chamber with a base pressure on ~3 × 10^−10^ mbar for in situ characterization. Some samples were transferred through a vacuum suitcase to STM (LT, Omicron) for low temperature characterization. STM images were acquired at all temperatures using W-tips.

### ARPES measurements

In situ ARPES measurements were performed at Beamline 10.0.1, Advanced Light Source, Lawrence Berkeley National Laboratory. ARPES data were acquired by Scienta R4000 electron analyzer. The system energy resolution and the angular resolution were set to 12 meV and 0.2°. The potassium was evaporated from a SAES Getters alkali metal dispenser to surface dope the thin film samples at the temperature of 10 K. Additional ARPES measurements were performed at the 03U beamline of the Shanghai Synchrotron Radiation Facility (SSRF) equipped with Scienta-Omicron DA30 electron analyzer. The angular and the energy resolutions were set to 0.2° and 8–20 meV (dependent on the selected probing photon energy).

### trARPES measurements

The grown films were transferred into the trARPES load-lock (pressure 1 × 10^−9 ^Torr) through a vacuum suitcase with a base pressure of 3 × 10^−10 ^Torr. The trARPES measurements at Stanford were based on a Ti: Sapphire regenerative amplifier operating at a repetition rate of 300 kHz. We used 1.5 eV linearly polarized IR pulse to excite the sample and used 6.0 eV UV pulse to probe the transient populations of the occupied and unoccupied band structure at a variable delay time. The overall time resolution of 70 ± 5 fs was extracted from cross-correlations of pump and probe pulses, and t_0_ refers to both pulses overlapping in time. The beam profiles for IR and UV were 69 × 10^2^ μm^2^ and 23 × 30 μm^2^, respectively. The photo-emitted electrons were collected by a Scienta R4000 analyzer in an ultrahigh vacuum with a base pressure less than 7 × 10^−11^ Torr. The energy resolution was 40 meV. During the measurement, the sample temperature was maintained at 12 K.

### Theoretical calculation

The theoretical result as shown in the left panel of Fig. 4b was calculated using the following method: Firstly, the structural relaxation was performed for the 2 × 2 supercell of the ZrTe_2_ structure using density functional theory calculation with generalized gradient approximation (GGA) functional implemented in quantum espresso^42^. The relaxed structure showed the CDW pattern. Secondly, the effective microscopic Hamiltonian for the CDW supercell, including the Zr 3*d* and Te 2*p* orbitals, was obtained using the Wannier downfolding, implemented in Wannier90^43^. An energy shift of 1 eV has been implemented on the on-site energies of the Zr 3*d* orbitals, in order to make the bandgap of the simulations consistent with the experiment. Thirdly, the band structure was calculated for the obtained tight-binding model Hamiltonian, with the wavefunctions projected on the primitive Brillouin zone associated with the original unit cell. Red and blue weights represent the orbital content for Zr and Te respectively.

The theoretical result as shown in the right panel of Fig. 4b was calculated using the following method: Firstly, the density functional theory calculation using quantum espresso^42^ and the Wannier downfolding for the Zr 3*d* and Te 2*p* orbitals using Wannier90^43^ were implemented for the unit cell of ZrTe_2_, obtaining the effective microscopic Hamiltonian. An energy shift of 1 eV has been applied to the on-site energies of the Zr 3*d* orbitals to make consistent with the experimental band structure. Secondly, the band structure for the primitive Brillouin zone was obtained using the tight-binding model Hamiltonian. Thirdly, the excitonic interactions were added between the top of the first (or second) valence band and the bottom of the first conduction band with momentum q difference. The Hamiltonian could be written as:

$$H\,=\,{H}_{{tight}-{binding}}\,+\,\mathop{\sum}\limits_{q\,=\,q1,\,q2,q3}\mathop{\sum}\limits_{k}{{\triangle }_{1}c}_{k,v1}^{+}{c}_{k\,-\,q,c1}\,+\,{{\triangle }_{2}c}_{k,v2}^{+}{c}_{k\,-\,q,c1}\,+\,h.c.$$

in which ${H}_{{tight}-{binding}}$ is the tight-binding Hamiltonian obtained using Wannier90, $q1\,=\,\left(0.0,-0.5\right),\,q2\,=\,\left(0.5,\,0.0\right),q3\,=\,(-0.5,0.5)$, $v1$ and $v2$ represent the first (top) and the second valence bands respectively, $c1$ represents the first conduction band, ${\triangle }_{1}\,=\,0.07\,{eV}$ and ${\triangle }_{2}\,=\,0.005\,{eV}$ in the calculation. The obtained band structure of this Hamiltonian was plotted on the right panel of Fig. 4b. Red and blue weights represent the orbital content for Zr and Te respectively.

## Supplementary information

Supplementary Information

## Data Availability

All the data that support the findings of this study are available from the corresponding author on reasonable request.
