# Supplementary material for: Signatures of the exciton gas phase and its condensation in monolayer 1T-ZrTe2
Source: Nat Commun. 2023 Feb 27;14:1116. doi: 10.1038/s41467-023-36857-7 (PMC9971207; doi:10.1038/s41467-023-36857-7)
Supplement: Supplementary file 1 — Supplementary Information [file 41467_2023_36857_MOESM1_ESM.pdf]

Signatures for the exciton gas phase and its condensation in monolayer 1T-$ZrTe_2$

Yekai Song[1,2†], Chunjing Jia[3,4†], Hongyu Xiong[3,5,6], Binbin Wang[7], Zhicheng Jiang[1], Kui Huang[7], Jinwoong Hwang[5,8,9], Zhuojun Li[1,2], Choongyu Hwang[10], Zhongkai Liu[7], Dawei Shen[1,11], Jonathan A. Sobota[3,5], Patrick Kirchmann[3,5], Jiamin Xue[7], Thomas P. Devereaux[3,5], Sung-Kwan Mo[8*], Zhi-Xun Shen[3,5*], Shujie Tang[1,2*]

[1]State Key Laboratory of Functional Materials for Informatics, Shanghai Institute of Microsystem and Information Technology, Chinese Academy of Sciences, Shanghai 200050, China.

[2]2020 X-Lab, Shanghai Institute of Microsystem and Information Technology, Chinese Academy of Sciences, Shanghai 200050, China.

[3]Stanford Institute for Materials and Energy Sciences, SLAC National Accelerator Laboratory, 2575 Sand Hill Road, Menlo Park, California 94025, USA.

[4]Department of Physics, University of Florida, Gainesville FL 32611, USA.

[5]Geballe Laboratory for Advanced Materials, Departments of Physics and Applied Physics, Stanford University, Stanford, California 94305, USA.

[6]Key Laboratory for Power Machinery and Engineering of MOE, School of Mechanical Engineering, Shanghai Jiao Tong University, Shanghai 200240, PR China.

[7]School of Physical Science and Technology, ShanghaiTech University, Shanghai 201210, China.

[8]Advanced Light Source, Lawrence Berkeley National Laboratory, Berkeley, California 94720, USA.

[9]Department of Physics, Kangwon National Univerisity, Chuncheon, 24341, Korea

[10]Department of Physics, Pusan National University, Busan 46241, Korea.

[11]National Synchrotron Radiation Laboratory, University of Science and Technology of China, 42 South Hezuohua Road, Hefei, Anhui 230029, China

*Corresponding authors. Email: skmo@lbl.gov, zxshen@stanford.edu, tangsj@mail.sim.ac.cn

Contents:

Supplementary Notes 1-6

Supplementary Figs. 1-6

Supplementary References

Supplementary Note 1: Core level

The angle-integrated core level photoemission spectrum (Supplementary Fig. 1) exhibits the characteristic peaks of Zr 4*p* and Te 4*d* orbitals with no splitting observed.

Supplementary Note 2: Fitting the EDC peaks

In order to extract the exact band gap size, one needs to get the peak position of the conduction band bottom and the valence band top under different temperatures. We used Lorentz peaks to fit the energy distribution curves (EDCs).

At low temperature (CDW state), the two distinguishable peaks below E_F from the EDCs at the M point correspond to the folded first valence band and the original conduction band respectively. The Lorentz peaks of the fit are plotted as the solid green and blue lines, and the fitted peak is the solid black lines, which fit the original EDCs (red dots) very well (Supplementary Fig. 2a). The direct band gap is determined by the peak position of the two Lorentz peaks, marked as the solid red circles in Fig. 2d.

At high temperature (normal state), it is hard to determine whether the folded valence merges into the conduction band or disappears. Thus, two different fitting strategies were employed and the results show an identical trend. On the one hand, we continued to use two Lorentz peaks to fit the single peak observed, the fitted single peak (solid black line) can also be consistent with the original EDC (Supplementary Fig. 2b). The direct gap determined by the peak position of the two Lorentz peaks are marked as the hollow red circles in Fig. 2d. On the other hand, the indirect gap sizes are determined by the single peak at the M point as the conduction band bottom (Supplementary Fig. 2c) and the valence band top at the Γ point together, marked as the solid blue circles in Fig. 2d.

Supplementary Note 3: Laser ARPES Measurement

To further confirm the nature of the band top of the first valence band at the Γ point in the normal state (Fig. 3c), we used laser ARPES to study the band structure at the Γ point near E_F at 300 K, as shown in Supplementary Fig. 3. It can be clearly observed that the first valence band is well below the E_F, and its dispersion and the spectral weight transfer are the same as the one in the main text.

Supplementary Note 4: The interaction-free band dispersion

The interaction-free band dispersion has been calculated using DFT with the PBE method within a semi-local approximation. In the low-temperature CDW state (Supplementary Fig. 4a), the band structure observed experimentally is basically consistent with the interaction-free band dispersion (blue dotted line) except for the ones near E_F, which has a valence band with the flat band top at the Γ point, a folding valence band and a direct gap at the M point. For the interactions-suppressed state (Fig. 3e), the band structure near E_F is renormalized to be more consistent with the PBE calculations. Firstly, the first valence band at the Γ point transforms from a flat band top to a nearly linear dispersion; Secondly, the first valence band and the conduction band overlaps with each other below E_F, although the overlap is not as significant as the DFT result. In addition, a faint conduction band at the Γ point is observed to be almost in touch with the valence band (Supplementary Fig. 4b), which is consistent with the calculated and previously reported band structure[1].

Supplementary Note 5: Doping dependent ARPES Measurement

To suppress the interaction at the CDW state, we performed potassium (K) surface doping on the sample, which injects electrons to a sample and raise chemical potential. Supplementary Fig. 5 shows the evolution of electronic structure with the increasing amount of K doping. The conduction band at the M point gradually moves towards deeper binding energy, and carrier

density increases accordingly. At lower carrier density, the evolution of the band gap and the folding of the second valence band is similar to one caused by temperature change.

Supplementary Note 6: Atomically resolved STM

A 2×2 superlattice is observed by the atomically resolved STM image and its FFT image at T = 100 K (Supplementary Fig. 6a), indicating the existence of CDW order and this temperature is below T_c. And the 2×2 superlattice is disappeared at T = 150 K (Supplementary Fig. 6b), which indicates that this temperature is above T_c.

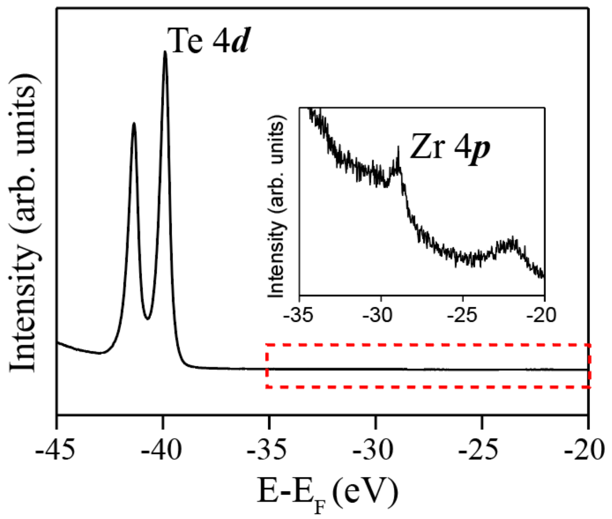

Supplementary Fig. 1 | Core level spectrum of the monolayer 1T-$ZrTe_2$ film.

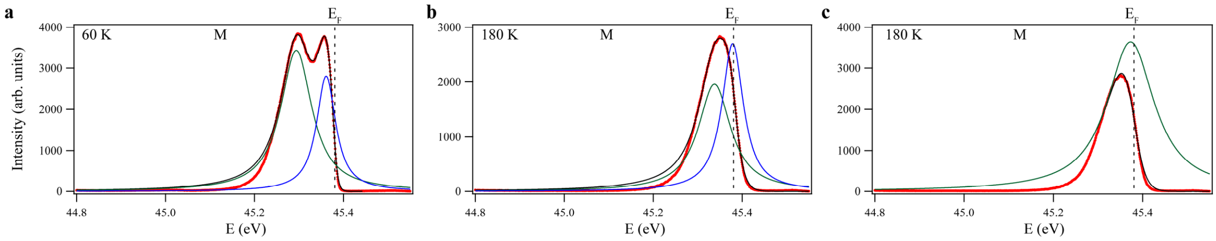

Supplementary Fig. 2 | Details of the EDC fitting. a. The EDC at the M point at 60 K are fitted with two Lorentz peaks. **b and c.** Two and one Lorentz peaks fitting strategy for the EDC at the M point at 180 K, respectively. The solid green and blue lines are the Lorentz peaks, and the solid black lines are the final fitting result. The red dots are the original EDCs.

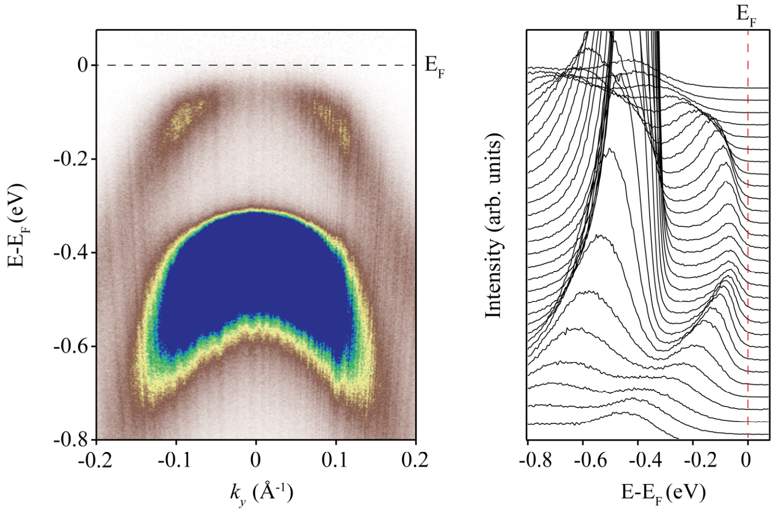

Supplementary Fig. 3 | The band structure at the Γ point measured at 300 K using laser light with 7 eV. The E_F location was determined by measuring the metallic edge of a polycrystalline gold reference. The ARPES data was divided by the Fermi-Dirac function at 300 K.

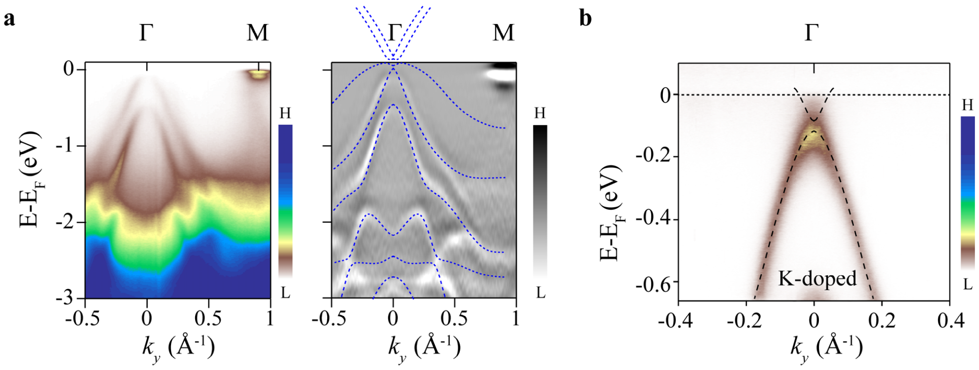

Supplementary Fig. 4 | Comparisons between theoretical and experimental electronic structure. a. The band structure measured by ARPES along the Γ-M direction at 15 K and the corresponding second derivative plot. The calculations are illustrated by the blue dotted lines for comparison. **b.** ARPES data at the Γ point taken from surface K-doped sample. The black dotted lines serve as the guide lines.

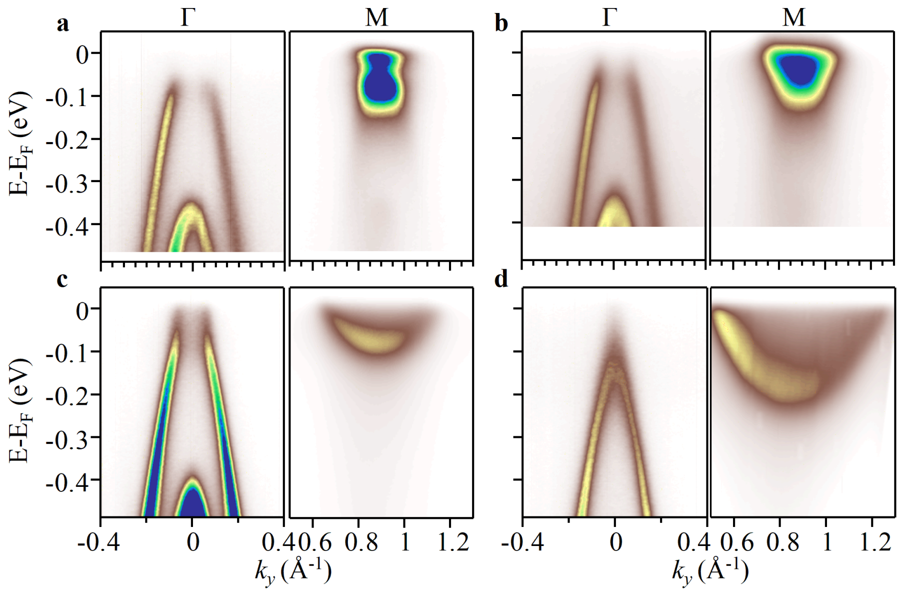

Supplementary Fig. 5 | Evolution of electronic structure with increasing amount of K doping. **a.** The band structure in the pristine sample. **b-d.** The band structure under different amount of K doping with a carrier density of 0.006 e$^-$/Zr atom (b), 0.05 e$^-$/Zr atom (c) and 0.14 e$^-$/Zr atom (d).

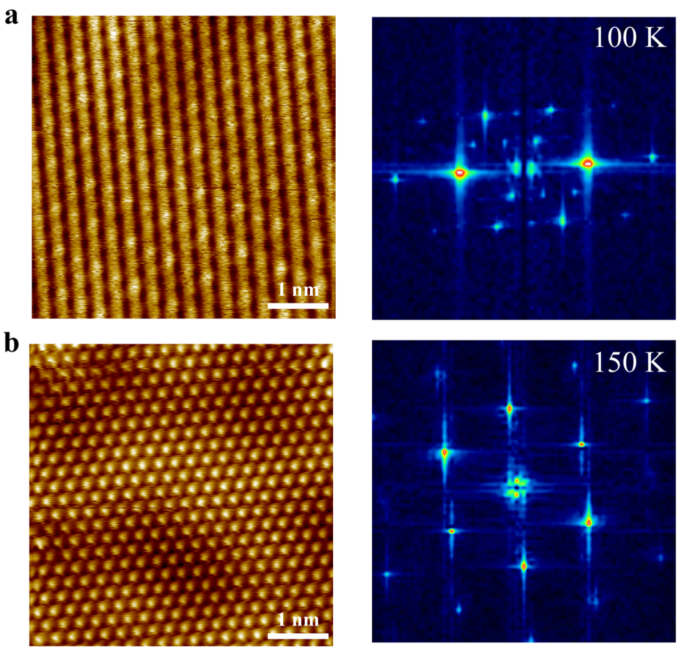

Supplementary Fig. 6 | Temperature-dependent STM results. a. Atomically resolved STM image of 5 nm × 5 nm and corresponding FFT image at 100 K [V_{bias} = -50 mV, I = 50 pA]. **b.** Atomically resolved STM image of 5 nm × 5 nm and corresponding FFT image at 150 K [V_{bias} = -50 mV, I = 250 pA].

Supplementary References

1. Tsipas, P. *et al.* Massless Dirac Fermions in $ZrTe_2$ Semimetal Grown on InAs(111) by van der Waals Epitaxy. *Acs Nano* **12**, 1696-1703 (2018)
